# Burden of respiratory syncytial virus hospitalisation among infants born at 32–35 weeks' gestational age in the Northern Hemisphere: pooled analysis of seven studies

**DOI:** 10.1017/S0950268820001661

**Published:** 2020-08-17

**Authors:** M. Lanari, E.J. Anderson, M. Sheridan-Pereira, X. Carbonell-Estrany, B. Paes, B.S. Rodgers-Gray, J. R. Fullarton, E. Grubb, M. Blanken

**Affiliations:** 1 Pediatric Emergency Unit, Department of Medical and Surgical Sciences, S. Orsola University Hospital, Bologna, Italy; 2 Departments of Pediatrics and Medicine, Emory University School of Medicine, Atlanta, GA, USA; 3 Department of Paediatrics, Trinity College and Coombe Women and Infants University Hospital, Dublin, Ireland; 4 Neonatology Service, Hospital Clinic, Institut d'Investigacions Biomediques August Pi Suñer (IDIBAPS), Barcelona, Spain; 5 Department of Pediatrics (Neonatal Division), McMaster University, Hamilton, Ontario, Canada; 6 Strategen Limited, Winchester, UK; 7 Health Economics and Outcomes Research, AbbVie Inc, North Chicago, Illinois, USA; 8 Division of Pediatric Immunology and Infectious Diseases, University Medical Center Utrecht, Utrecht, The Netherlands

**Keywords:** Epidemiology, Lower respiratory tract infection, Moderate-to-late preterm infants, Respiratory support, Respiratory syncytial virus, RSV hospitalisation

## Abstract

To provide comprehensive information on the epidemiology and burden of respiratory syncytial virus hospitalisation (RSVH) in preterm infants, a pooled analysis was undertaken of seven multicentre, prospective, observational studies from across the Northern Hemisphere (2000–2014). Data from all 32^0^–35^6^ weeks' gestational age (wGA) infants without comorbidity were analysed. RSVH occurred in 534/14 504 (3.7%) infants; equating to a rate of 5.65 *per* 100 patient-seasons, with the rate in individual wGA groups dependent upon exposure time (*P* = 0.032). Most RSVHs (60.1%) occurred in December–January. Median age at RSVH was 88 days (interquartile range (IQR): 54–159). Respiratory support was required by 82.0% of infants: oxygen in 70.4% (median 4 (IQR: 2–6) days); non-invasive ventilation in 19.3% (median 3 (IQR: 2–5) days); and mechanical ventilation in 10.2% (median 5 (IQR: 3–7) days). Intensive care unit admission was required by 17.9% of infants (median 6 days (IQR: 2–8) days). Median overall hospital length of stay (LOS) was 5 (IQR: 3–8) days. Hospital resource use was similar across wGA groups except for overall LOS, which was shortest in those born 35 wGA (median 3 *vs.* 4–6 days for 32–34 wGA; *P* < 0.001). Strategies to reduce the burden of RSVH in otherwise healthy 32–35 wGA infants are indicated.

## Introduction

Respiratory syncytial virus (RSV) remains a leading cause of lower respiratory tract infection (LRTI) in early childhood [1], causing over 375 000 hospitalisations (respiratory syncytial virus hospitalisation (RSVHs)) *per* year in children <5 years in high-income countries [2]. RSVHs are concentrated during seasonal outbreaks – the winter months in temperate countries – and place a major demand on neonatal and paediatric services at this time of year [3]. Certain children, such as those with co-morbidities, including congenital heart disease and chronic lung disease, immunodeficiency and those born prematurely, are known to be particularly vulnerable to severe RSV LRTI [4–6].

An important group that has generated much debate comprises those children born 32–35 weeks' gestational age (wGA), in terms of their level of risk for RSVH compared to those born at lower and higher wGA and the cost-effective deployment of prophylaxis with palivizumab [7]. This is reflected in international guidelines, with some, such as from the American Academy of Pediatrics [8, 9], not recommending RSV prophylaxis in 32–35 wGA infants, while others, such as from Spain and Italy [10, 11], recommending the use of risk factors to guide prophylaxis in 32–35 wGA infants at highest risk. Palivizumab currently represents the only licensed and effective prophylaxis for preventing RSVH in high-risk infants, including those born 32–35 wGA, although several promising monoclonal antibody candidates could become available in the next few years [12]. A comprehensive understanding of the risk and burden of RSVH in otherwise healthy 32–35 wGA infants is important to help guide preventive interventions.

A number of prospective studies have assessed the burden of RSVH in children born 32–35 wGA within Northern Hemisphere countries [13–19]. To provide a greater understanding of the burden of RSVH across the Northern Hemisphere, we previously analysed pooled data from seven studies that focused on 33–35 wGA infants born and hospitalised during the RSV season [20]. In total, 267 out of 7820 (3.4%) 33–35 wGA infants were hospitalised for RSV during the RSV season, with an overall rate of 4.5 *per* 100 patient-seasons. The median hospital length of stay (LOS) was 6 days, and 22% required intensive care unit (ICU) admission for a median of 8 days. Supplemental oxygen support was required by 70.4% of infants for a median of 5 days and 12.7% required mechanical ventilation for a median of 5 days [20].

Herein we analysed those born at 32 wGA, stratified results across individual wGA, and assessed those born and hospitalised outside of the RSV season.

## Methods

### Included studies

The selection of studies comprising the pooled dataset has been described previously [20]. Briefly, studies of RSVH in 32–35 wGA infants (defined as 32 weeks and 0 days (32^0^) to 35 weeks and 6 days (35^6^)) were identified *via* a systematic search of PubMed and EMBASE from January 1998 to January 2015. Study selection criteria included a multicentre, observational, prospective design; inclusion of >1000 preterm infants; and laboratory-confirmed RSV infection in all subjects (direct immunofluorescence assay, rapid antigen/enzyme membrane testing, rapid shell viral detection or polymerase chain reaction (PCR)-based assays), who were hospitalised with an LRTI. Studies where >15% of infants received palivizumab prophylaxis were excluded (to standardise and to avoid potential confounding) and only one study (most recent) from an individual country was included (to limit data to one representative study from each country). The overall aim was to include large, high-quality, cross-sectional studies that, when combined, provide an overview of the burden of RSV generalisable across the Northern Hemisphere.

The original review comprised seven studies (spanning 2000–2014) [20], and no additional reports were identified when the searches were updated to 31 January 2020. The seven studies were as follows: Risk *F*actors *L*inked to Respiratory Syncytial Virus *I*nfection Requiring Hospitalization in *P*remature Infants Study (FLIP-2, Spain) [13]; RISK Study (the Netherlands) [14]; *P*ediatric *I*nvestigators *C*ollaborative *N*etwork on *I*nfections in *C*anada (PICNIC, Canada) [15]; RSV *P*reterm *R*isk *E*stimation *M*easure for RSV Hospitalization in *I*reland (RSV-PREMI, Ireland) [16]; *I*talian National *B*irth *C*ohort (IBC, Italy) [17]; *R*SV Respiratory *E*vents among *P*reterm Infants *O*utcomes and *R*isk *T*racking (REPORT, USA) [18]; and *P*redictors Associated with RSV H*O*spitalization in *N*onprophylaxed, Premature *I*nfants (PONI, multinational) [19].

### Cohort selection

All 32^0^–35^6^ wGA infants (≤1 year) were included in this analysis except: those with a comorbidity associated with an increased risk of RSVH (e.g. Down syndrome, immunodeficiency, congenital heart disease, bronchopulmonary dysplasia); those who had received palivizumab prophylaxis; and those with an RSV-negative respiratory-related hospitalisation. Gestational age was determined within each study based on standard criteria, namely ultrasonographic dating and/or the time between the first day of the last menstrual period and delivery date.

### Pooled analysis

The individual datasets from the seven studies were previously shown to be homogeneous when analysed in 33–35 wGA infants born and hospitalised during the RSV season [20]. Predicated on this, for the current analysis, data were amalgamated and analyses performed on the combined dataset without weighting. All seven studies contained data on infants born 33–35 wGA, while four (FLIP-2, PREMI, REPORT and RISK) also included 32 wGA infants (Table 1). Age at first RSVH and distribution of RSVHs by month were calculated using data from studies that had year-round surveillance (FLIP-2, IBC, PREMI and RISK), while results stratified by wGA used data only from those studies that included 32 wGA infants (FLIP-2, PREMI, REPORT and RISK).

**Table 1.** Overview of included studies

Study	Country	Time span	Follow-up duration	Included wGA	*N*	RSV season
PICNIC	Canada	2000–2002	1 month post-RSV season	33^0^–35^6^	1832	1 November–30 April
FLIP-2	Spain	2005–2007	End of May	32^1^–35^0^	5441	1 October–30 April
RISK	The Netherlands	2008–2012	1 year of age	32^1^–35^6^	2421	1 October–31 March
REPORT	USA	2009–2011	End of May	32^0^–35^6^	1642	1 November–31 March
IBC	Italy	2009–2013	1 year of age	33^0^ +	2210	1 November–31 March
PREMI	Ireland	2011–2014	1 year of age	32^0^–36^6^	1807	1 October–31 March
PONI^a^	Europe, Middle East, North America, Asia	2013–2014	End of April	33^0^–35^6^	2390	1 October–30 April

RSV, respiratory syncytial virus; wGA, weeks' gestational age (superscript digit indicates days).

a PONI countries: Austria, Bahrain, Czech Republic, Egypt, Estonia, France, Jordan, Korea, Latvia, Lebanon, Lithuania, Mexico, Norway, Oman, Portugal, Russia, Saudi Arabia, Slovakia, Slovenia, Sweden.

### Outcomes

RSVH frequency was expressed as both an incidence rate and as a rate *per* 100 patient-seasons, with the latter adjusted for length of exposure during the RSV season for the full cohort (NB in our previous analysis restricted to 33–35 wGA infants born and hospitalised within the RSV season +1 month, the exposure rate used was for RSVH cases only [20]). Season start and end dates were taken from each study and confirmed with the respective study leads, and ranged between 1 October and 30 April (Table 1). RSVHs were further stratified by month during the RSV season and by wGA. RSVH in relation to chronological age was also assessed split by those born during and outside the RSV season.

The following outcomes related to RSVHs were assessed: requirement for and duration of oxygen support; requirement for and duration of other non-invasive respiratory support/ventilation (defined as the use of high-flow nasal cannula, continuous positive airway pressure and/or nasal intermittent positive pressure ventilation/non-invasive positive pressure ventilation); requirement for and duration of mechanical ventilation (defined as any intervention that included intubation or extracorporeal membrane oxygenation); admission to and LOS in ICU; and overall hospital LOS.

### Statistical analysis

LOS and duration of respiratory support required were expressed as a median with interquartile range (IQR). Length of follow-up was presented as a mean plus standard deviation (SD). RSVH incidence rates were compared using the Kruskal–Wallis test and Spearman (non-parametric) correlation. Differences in hospital resource use by wGA were assessed using the Kruskal–Wallis test. Analyses of partial seasonal exposure in relation to RSVH across gestational age groups were carried out using Cox regression. Analyses were performed using SPSS for Windows version 15.0 (SPSS Inc, Chicago, IL, USA), Microsoft Access SQL (Microsoft, Redmond, WA, USA) and/or Microsoft Access/Excel VBScript (Microsoft). A *P*-value <0.05 was considered statistically significant.

## Results

### Pooled dataset

**Fig. 1.**
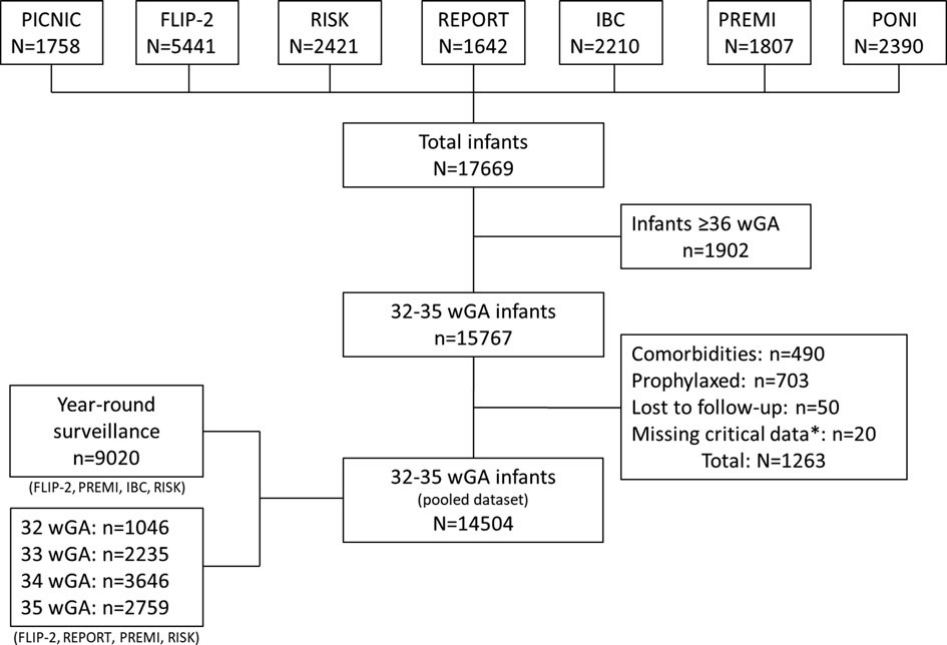
Derivation of pooled dataset.

In total, the pooled analysis included 14 504 infants born between 32^0^ and 35^6^ wGA, for which year-round data were available for 9020 (Fig. 1). For the analyses split by wGA (*n* = 9686) there were: 1046/9686 (10.8%) infants born at 32 wGA, 2235/9686 (23.1%) at 33 wGA, 3646/9686 (37.6%) at 34 wGA and 2759/9686 (28.5%) at 35 wGA.

wGA, weeks' gestational age; *9 cases which were not hospital confirmed, 11 where there was no record of wGA.

### Epidemiology of RSVH

#### Overall

**Fig. 2.**
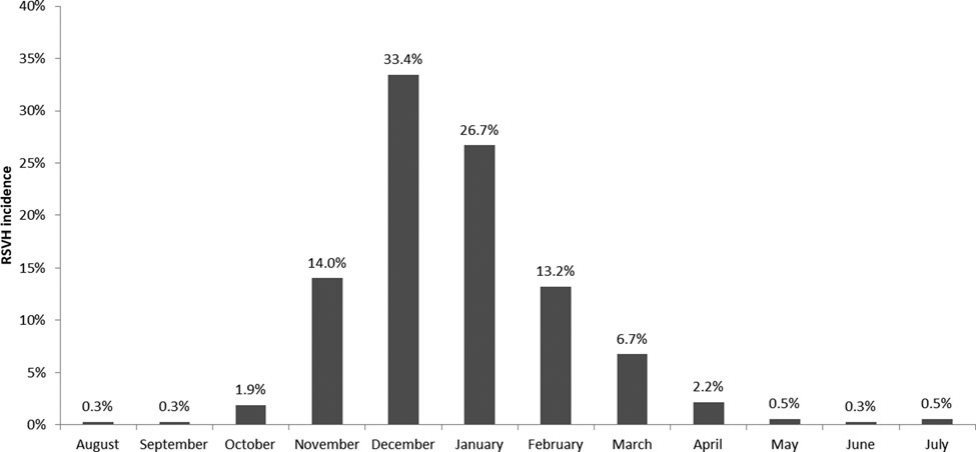
Distribution of RSVH incidence by month*

Of the 14 504 infants, 534 had a confirmed RSVH, representing an incidence of 3.7%. The corresponding rate *per* 100 patient-seasons was 5.65. December and January accounted for over half of RSVHs (60.1%), with 94.1% of RSVHs occurring between November and March (Fig. 2); across a mean follow-up of 204.1 (SD: 104.9) days. In total, 2.4% (9/371) of RSVHs occurred outside the RSV season. RSVH incidence rates were similar for those born within and outside the RSV season (4.3% *vs.* 3.9%, respectively; *χ*^2^: *P* = 0.25). Overall, 42.3% of RSVHs (157/371) occurred in infants born outside the RSV season.

RSVH, respiratory syncytial virus hospitalisation. *Data from FLIP-2, IBC, PREMI and RISK (studies with year-round data capture). Total RSVHs: 371.

Median age at RSVH was 88 (IQR: 54–159) days, with approximately half of RSVHs (52.6%; 195/371) occurring in infants <3 months old and four-fifths occurring in infants ≤6 months old (81.7%; 303/371). For those born within and outside the RSV season, the median age at RSVH was 62 (IQR: 40–96) days and 143 (IQR: 94–188) days, respectively.

#### Analysis by wGA

RSVH incidence tended to increase with decreasing wGA at birth, although this did not reach statistical significance: 4.0% for those born 35 wGA; 4.0% for 34 wGA; 4.7% for 33 wGA; and 5.0% for 32 wGA (*χ*^2^: *P* = 0.09; Spearman: *P* = 0.10). However, when converted to a rate *per* 100 patient-seasons, the difference between wGA groups was significant (Spearman: *P* = 0.03): 35 wGA 5.66; 34 wGA 6.14; 33 wGA 7.29; and 32 wGA 7.84. The length of seasonal exposure to RSV also increased with increasing wGA (Spearman: *P* = 0.003). Cox regression analysis showed an upward trend in mean seasonal exposure rates amongst RSVH cases between 32 wGA (0.638) and 35 wGA (0.707) and the Wald statistic for seasonal exposure as a predictor of change in hazard for RSVH was highly significant (*P* < 0.001) in every group. The proportion of RSVHs outside the RSV season did not differ significantly with wGA, albeit there was a rising rate with increasing wGA (32 wGA: 0.0%; 33 wGA: 2.0%; 34 wGA: 2.3%; 35 wGA: 2.7%; Spearman: *P* = 0.36). Median age at RSVH did not significantly differ across wGA groups (Spearman: *P* = 0.21), although infants born at 32 wGA (118 (IQR: 64–195) days) seemed to have an extended period of risk compared to those born at ³33 wGA (33 wGA: 82 (IQR: 55–153) days; 34 wGA: 91 (IQR: 51–154) days; 35 wGA: 89 (IQR: 47–159)).

### Healthcare resource use during RSVH

#### Overall

Four out of five infants (82.0%; 260/317) required some form of oxygen therapy or respiratory support during their RSVH; 70.4% required supplemental oxygen, 19.3% non-invasive ventilation and 10.2% mechanical ventilation (Table 2). Supplemental oxygen was used for a median of 4 (IQR: 2–6) days, non-invasive ventilation for 3 (IQR: 2–5) days and mechanical ventilation for 5 (IQR: 3–7) days. Of the cases, 17.9% were admitted to the ICU for a median of 6 (IQR: 2–8) days. Overall hospital LOS was for a median of 5 (IQR: 3–8) days.

**Table 2.** Healthcare resource use during RSVH

Resource	Outcome					32–35 wGA comparison
	32–35 wGA^a^	32 wGA^b^	33 wGA^b^	34 wGA^b^	35 wGA^b^	*P*-value
Oxygen support						
Requirement, *n*/*N* (%)	235/334 (70.4%)	29/38 (76.3%)	49/66 (74.2%)	60/92 (65.2%)	44/63 (69.8%)	0.092
Duration, median days (IQR)	4 (2–6) days	4 (2–6) days	3 (2–5) days	4 (2–7) days	3 (1–7) days	0.721
Non-invasive respiratory support/ventilation						
Requirement, *n*/*N* (%)	21/109 (19.3%)	0/9 (0.0%)	3/14 (21.4%)	2/6 (33.3%)	2/18 (11.1%)	0.287
Duration, median days (IQR)	3 (2–5) days	N/A	5 (4–6) days	7 (2–11) days	4 (2–6) days	0.815
Mechanical ventilation						
Requirement, *n*/*N* (%)	47/460 (10.2%)	4/50 (8.0%)	9/99 (9.1%)	14/137 (10.2%)	10/96 (10.4%)	0.970
Duration, median days (IQR)	5 (3–7) days	7 (4–11) days	6 (4–6) days	9 (3–13) days	6 (3–7) days	0.581
ICU						
Requirement, *n*/*N* (%)	60/335 (17.9%)	7/38 (18.4%)	7/66 (10.6%)	18/92 (19.6%)	9/64 (14.1%)	0.280
Duration, median days (IQR)	6 (3–8) days	7 (4–12) days	5 (1–9) days	6 (3–9) days	3 (2–7) days	0.119
Overall length of stay, median days (IQR)	5 (3–8) days	4 (3–7) days	5 (3–8) days	6 (4–9) days	3 (2–6) days	*P* < 0.001

ICU, intensive care unit; IQR, interquartile range; N/A, not applicable.

N represents all those with available data for specific variables in the pooled dataset.

a Pooled data from: FLIP-2, IBC, PREMI, RISK, PONI, REPORT and PICNIC.

b Pooled data from: FLIP-2, PREMI, REPORT and RISK.

#### Analysis by wGA

The percentages of infants requiring any form of respiratory support were highest in those born most premature, although results were not significantly different across wGA groups: 59.6% (31/52) for those born 32 wGA; 50.5% (53/105) for 33 wGA; 44.1% (64/145) for 34 wGA; and 43.6% (48/110) for 35 wGA (Kruskal–Wallis: *P* = 1.000; Spearman: *P* = 0.06). None of the other hospital resource variables differed significantly between 32, 33, 34 and 35 wGA infants except the median duration of overall hospital LOS, which was lower (*P* < 0.001) for those born 35 wGA (3 (IQR: 2–6) days) after a progressive increase in stay from 32 to 34 wGA (32: 4 (3–7) days; 33: 5 (3–8) days; 34: 6 (4–9) days).

## Discussion

This pooled analysis of 14 504 infants provides a comprehensive evaluation of the epidemiology and burden of RSVH in 32–35 wGA infants in the Northern Hemisphere. The overall incidence of RSVH was 3.7% and the overall rate *per* 100 patient-seasons was 5.65, with over half (60.1%) of all RSVHs occurring in December and January. While the asserted start and end dates of the RSV season varied by country (and sometimes within the country) and year-to-year, the main at-risk period spanned from November through March (94.1% of all RSVHs).

While RSVH incidence did not significantly differ with wGA (*P* = 0.10), the rate *per* 100 patient-seasons did significantly increase with decreasing wGA (35 wGA 5.66; 34 wGA 6.14; 33 wGA 7.29; 32 wGA 7.84; *P* = 0.032). Other studies, such as those from Denmark [21] and the USA [22], have reported increasing RSVH incidence rates with decreasing wGA. A Dutch community-based cohort study reported that overall RSVH rates of 32–36 wGA infants (*n* = 964, 3.9%) were greater than full-term infants (38–42 wGA; *n* = 572, 1.2%; relative risk (RR) 3.2, 95% CI 1.4–7.1), but equal to <32 wGA infants (*n* = 524; 3.9% *vs.* 3.2%, RR 1.2, 95% CI 0.7–2.1) [23]. Our study suggests that a key factor when comparing RSVH rates across wGA groups is the length of exposure to RSV, with more premature infants in our analysis requiring a shorter exposure time to be admitted for LRTI (Spearman: *P* = 0.003; Cox: *P* < 0.001).

The median age at RSVH was approximately 3 months (88 days), with half (53%) of all RSVHs occurring in those <3 months of age and four-fifths in those ≤6 months of age (82%). The median age at RSVH did not differ significantly across wGA (*P* = 0.21), although infants born 32 wGA appeared to have a longer at-risk period (median 118 days) than those born 33–35 wGA (82–91 days). Similar results were reported in the US SENTINEL 1 study, where the median age at RSVH was 3 months for 29–32 wGA infants and 2 months for 33–35 wGA infants [22]. Among 29–32 wGA infants, 55% of RSVHs occurred in those <4 months of age, and among 33–35 wGA infants, 55% of RSVHs occurred in those <3 months of age [24]. In total, 78% of RSVHs in 29–35 wGA infants were in those <6 months old [25]. Another US study, including preterm and term infants, reported that 64% of RSVHs occurred in infants ≤5 months old [26]. The RSVH incidence rates in our study were similar whether the infants were born within or outside the RSV season (4.3% *vs.* 3.9%; *P* = 0.25), possibly reflecting younger chronological age in the former balanced against the increased duration of exposure in the latter.

RSVHs were associated with considerable resource use, with 82% of infants requiring some form of respiratory support, 10% mechanical ventilation for a median of 5 days and 18% ICU admission for a median of 6 days. Studies from the USA, including SENTINEL 1, have reported higher ICU admission rates of 40–50% in similar wGA infants [25, 27], albeit comparable ICU LOS (5–7 days) [25]. Overall hospital LOS was also comparable in our study to SENTINEL 1 (median 5 *vs.* 5–6 days [25], respectively). It has been reported that 33–35 wGA infants had significantly higher resource use during RSVH than those born ≤32 or ⩾36 wGA [27], though this has not been substantiated in other studies. Interrupted lung development during the critical period before 36 wGA and immunologic immaturity are potential contributors to an increased RSVH risk [28, 29]. In our study, hospital resource use was broadly similar across each wGA group, although 35 wGA infants, on average, spent less overall time in hospital than their younger wGA counterparts (median 3 *vs.* 4–6 days, respectively; *P* < 0.001). The costs associated with an RSVH episode in preterm infants have been estimated at a median $14 000–77 000 in the USA [22, 25] and €2000–4000 in Europe [30]. After RSVH discharge, 32–35 wGA infants continue to have substantial medical expenses [31, 32].

The main limitations of our pooled analysis were related to some salient differences in methodology/design of the included studies. Most notably, only four of seven studies included 32 wGA infants, although data were available on >1000 such infants. Other differences included the fact that the PICNIC study enrolled only infants born and hospitalised during the RSV season (+1 month) and variable lengths of follow-up (Table 1). Heterogeneity between datasets was previously reported for age at admission (non-parametric Levene test: *P* = 0.001) and duration of hospitalisation (non-parametric Levene test: *P* < 0.001) [20]. The studies spanned 2000–2014 and RSV diagnostic tests have evolved during this period, with the earlier studies predominantly using direct immunofluorescence assay, rapid antigen/enzyme membrane testing and rapid shell viral detection, whilst the later studies used more sensitive PCR-based assays. Differences in admission criteria and medical practice between countries as well as cultural factors, such as frequency and earlier enrolment in day care, a recognised risk factor for RSVH, might also have influenced RSV admission rates across studies. For these reasons, the pooled analysis was undertaken without weighting and the analyses by wGA and age restricted to those studies that included 32 wGA infants and year-round surveillance, respectively. Despite some differences, the methodologies of the seven studies were broadly similar overall in terms of being multicentre, observational, prospective studies involving 32–35 wGA infants. It is also a strength that for five of the seven studies (FLIP-2, RISK, PICNIC, REPORT and PONI), representing 86% (12 449/14 504) of the total study population, RSV testing was part of the protocol [13, 14, 15, 18, 19]. A high proportion (80%; 93/116) of LRTI cases were also RSV tested in PREMI (70% (65/93) positive) [16]. In the Italian Birth Cohort [17], a quarter (26%; 31/120) of cases were tested (84% (26/31) positive). Taken together, it can be seen that the pooled analysis provides an accurate representation of the true burden of RSVHs in otherwise healthy 32–35 wGA infants in the Northern Hemisphere.

## Conclusion

Our study provides comprehensive data on the epidemiology and burden of RSVH in 32–35 wGA infants in the Northern Hemisphere that can help inform healthcare policy on the deployment of preventive strategies, such as palivizumab. The use of a country-specific or customisable risk factor scoring tool to identify the highest risk infants [33] should be implemented, as recommended by recent expert consensus [34]. Such strategies, together with the advent of RSV vaccine(s) and the potential for newer extended half-life monoclonal antibodies, could help to alleviate the burden of RSV LRTI in these at-risk infants.

## Data Availability

Requests for access to the data that support this study should be made to the corresponding authors, XCE.
